# Smartphone-Based Activity Recognition Using Multistream Movelets Combining Accelerometer and Gyroscope Data

**DOI:** 10.3390/s22072618

**Published:** 2022-03-29

**Authors:** Emily J. Huang, Kebin Yan, Jukka-Pekka Onnela

**Affiliations:** 1 Department of Mathematics and Statistics, Wake Forest University, Winston-Salem, NC 27106, USA; 2 Department of Biostatistics, Epidemiology, and Informatics, University of Pennsylvania, Philadelphia, PA 19104, USA; Kebin.Yan@pennmedicine.upenn.edu; 3 Department of Biostatistics, Harvard University, Boston, MA 02115, USA; onnela@hsph.harvard.edu

**Keywords:** accelerometer, activity recognition, digital phenotyping, gyroscope, movelet, sensor, smartphone

## Abstract

Physical activity patterns can reveal information about one’s health status. Built-in sensors in a smartphone, in comparison to a patient’s self-report, can collect activity recognition data more objectively, unobtrusively, and continuously. A variety of data analysis approaches have been proposed in the literature. In this study, we applied the movelet method to classify the activities performed using smartphone accelerometer and gyroscope data, which measure a phone’s acceleration and angular velocity, respectively. The movelet method constructs a personalized dictionary for each participant using training data and classifies activities in new data with the dictionary. Our results show that this method has the advantages of being interpretable and transparent. A unique aspect of our movelet application involves extracting unique information, optimally, from multiple sensors. In comparison to single-sensor applications, our approach jointly incorporates the accelerometer and gyroscope sensors with the movelet method. Our findings show that combining data from the two sensors can result in more accurate activity recognition than using each sensor alone. In particular, the joint-sensor method reduces errors of the gyroscope-only method in differentiating between standing and sitting. It also reduces errors in the accelerometer-only method when classifying vigorous activities.

## 1. Introduction

Physical activity patterns correlate with one’s health status and can be used to obtain information about the individual’s health profile. For example, people recovering from major surgeries may move less than what is typical for them; the duration of the changed activity patterns can provide information about the patient’s recovery trajectory [1]. Similarly, an increase in purposeless movement (e.g., pacing and inability to sit still) may be a symptom of depression [2]. Thus, it may be beneficial to monitor the relevant daily activities of people at risk of developing health conditions. This kind of information has traditionally been gathered by having patients take surveys, complete interviews, or write diaries [3]. Although these self-reports, as firsthand accounts, are beneficial, they also have some limitations [4]. For example, self-reported data are often questioned due to their natural proclivities toward bias: patients may downplay certain tendencies because they like to be viewed as “normal”. Self-reports may overestimate exercise levels for a “good social image” [5]. Patients can also provide inaccurate reports unintentionally because human memory is prone to mistakes [6].

Researchers have sought to find new objective ways of collecting more reliable physical activity data to complement the self-reported data. The rise in smartphone adoption and usage offers a unique opportunity to revolutionize patient health status monitoring in research settings and clinical practice. Built-in smartphone sensors, such as the GPS, accelerometer, gyroscope, or magnetometer, can track location and movement continuously and unobtrusively. These *in situ* data can be collected to objectively quantify daily activities. Smartphone data collection does not require outfitting patients with additional instruments and, thus, can be conducted over long periods of time [7]. Smartphones are also widely accessible to the population. Based on surveys by the Pew Research Center, as of 2021, about 85% of U.S. adults own smartphones, which is almost 2.5 times the percentage from 10 years ago [8]. The field of digital phenotyping has emerged to take advantage of this new technological breakthrough and the vast amount of smartphone sensor data. Digital phenotyping is defined as the “moment-by-moment quantification of the individual-level human phenotype *in situ* using data from smartphones and other personal digital devices” [9]. This approach uses smartphones to capture high-throughput data to learn about cognitive, behavioral, and social phenotypes in free-living settings.

Human activity recognition (HAR) using smartphones has proliferated in recent years [10]. The first component of HAR is data collection, which requires careful thought about various questions, such as choosing the appropriate sensors, sampling frequency, study environment, and smartphone placement. Some studies use a single sensor [11,12], while other studies simultaneously utilize multiple sensors [13,14,15,16,17,18]. In our study, we used data collected from two sensors in the smartphone—the accelerometer and gyroscope.

The second component of HAR is data analysis. With improvements in technology, cost, and quality of data collection, the main challenge in HAR is shifting to data analysis, i.e., to extract the activities from the sensor data accurately and robustly [7,10,19]. In general, a given data analysis procedure can be divided into three steps: preprocessing, feature extraction, and activity classification [10]. Preprocessing prepares the data for the analysis at hand. For example, it might include removal of irrelevant high-frequency fluctuations (noise). The feature extraction step involves selecting and extracting representative features from the data. In activity classification, the extracted features are first associated with physical states or physical activities using statistical models. These models are then used to classify activities for new data.

Previous HAR studies have used a variety of feature extraction and activity classification techniques. A rapidly developing field is the application of deep learning, which automates both feature extraction and activity classification. Using multiple layers in the network, the deep learning procedure identifies optimal features from the raw data itself, without human intervention [20]. Some studies show that this approach can yield highly accurate results in activity classification [21,22,23]. However, there are limitations and challenges in the application. First, a vast amount of data is required to train a deep learning algorithm. Second, the model is usually used as a black box, and the extracted features from the multi-layered procedure can be difficult to interpret [20], resulting in difficulties in algorithm improvement.

A more traditional approach of data analysis is to view the data in short segments, referred to as windows. This approach allows us to examine the data directly and choose which features to extract through the most appropriate methods. Subsequently, a model may be constructed from training data to connect the selected features to activities. In this paper, we adopted the “movelet method” for feature extraction and activity classification, which was developed by Bai et al. [24] and later augmented by Huang and Onnela [11]. The movelet method is tailored to each individual patient by constructing a personal dictionary of windows for different types of activities from her/his training data. The patient’s activities are then inferred by comparing new data with the data in the dictionary [24]. The unique advantages of the movelet method are that it is intuitive, transparent, and personalized to each individual patient. The movelet method, in comparison to more sophisticated machine learning methods, only requires a small amount of training data (a few seconds per activity).

Some previous studies have used the movelet method to classify activities with a single sensor [11,24]. Bai et al. [24] analyzed data collected by a body-worn accelerometer. Huang and Onnela [11] applied the method to smartphone accelerometer data and separately to smartphone gyroscope data. The results showed that the smartphone accelerometer and gyroscope each had strengths in picking up different activities. In this study, we analyzed smartphone accelerometer data and gyroscope data jointly. Our hypothesis is that combined information from both acceleration and angular velocity would improve the accuracy of classification because the individual sensors capture different aspects of movement. The previous study by He et al. [25] used multiple accelerometers fixed to different parts of the body. They found improvements in classification accuracy using the integrated information from the multiple instruments. Although the smartphone is different from body-worn instruments, we expected its multiple sensors to provide similar benefits in improving classification accuracy. In comparison to multiple body-worn instruments, the smartphone has the advantage that it is compact, convenient to carry, and can be used over long time periods. In this paper, we present an extended version of the original movelet method that jointly incorporates smartphone accelerometer and gyroscope data. Moreover, we apply the method to our recent study and discuss the results. Our R code is provided on GitHub.

The paper is organized as follows. Section 2 describes the data set and presents our method for incorporating accelerometer and gyroscope data jointly in the movelet method. In Section 3, we present the results of applying this method to the study data set. We also compare the results to those from applying the movelet method to accelerometer data only and to gyroscope data only. Section 4 summarizes the results and discusses potential areas of future research.

## 2. Materials and Methods

### 2.1. Study Data Set

The data set used in this paper is from a study we conducted in 2018. The study included four participants. There were two female and two male participants, ranging in age from 27 to 54. Characteristics of the participants are provided in Table S1 of Huang and Onnela [11], including sex, height, weight, and dominant hand. For full disclosure, participant 1 is an author of this paper. Each participant had a study visit in which she/he performed a series of activities while wearing a study iPhone in the front right pants pocket and another study iPhone in the back right pants pocket. Throughout this paper, we focus on the front pocket phone, and refer to it as “the phone”.

In our study, we collected data from both the accelerometer and gyroscope sensors in the smartphone, with the phone placed in the front pants pocket. An accelerometer measures the acceleration of a phone along each of three orthogonal axes of a Cartesian coordinate system. The *x*-axis and *y*-axis are in the plane of the phone’s screen, with *x* pointing right and *y* pointing to the top of the phone. The *z*-axis points up through the phone, following the right hand rule. A gyroscope measures the angular velocity of a phone about three orthogonal axes. In previous HAR studies, a variety of sampling frequencies (samples per second) have been used (e.g., 1 Hz or even 100 Hz), commonly ranging between 20 and 30 Hz [10]. In our study, we sampled accelerometer and gyroscope data at a frequency of 10 Hz (i.e., 10 samples per second). The sampling frequency of 10 Hz was chosen because it is sufficient for capturing most daily activities.

Participants were observed separately. For each participant, her/his study visit consisted of two phases, training data collection and test data collection. During the training data collection, accelerometer and gyroscope data were recorded as the participant performed designated activities. These activities included walking, standing, ascending stairs, descending stairs, sitting, transitioning from sitting to standing (sit-to-stand), and transitioning from standing to sitting (stand-to-sit). In our analysis for each participant, we used 5 s of training data per activity for the activities of walking, standing, sitting, ascending stairs, and descending stairs. The training data for stand-to-sit used in the analysis came from one transition from standing to sitting. Analogously, the training data used for sit-to-stand was from one transition from sitting to standing. The duration of the training data for sit-to-stand and stand-to-sit were each shorter than 5 s because these activities are momentary transitions. The full protocol for the training data collection is provided in Huang and Onnela [11] (see Table 1 of their paper).

The test data collection included six steps, where the participant followed a prescribed course of activities on the Harvard Longwood campus. For example, the course in step 1 included walking, ascending stairs, standing, and descending stairs. The participant walked at different speeds in step 3, and ascended and descended a long staircase in step 6. A complete description of steps 1–6 in test data collection is provided in Huang and Onnela [11] (see Table 1 of their paper). The test data were collected in public spaces outdoors and indoors, not in a tightly controlled lab environment. We chose these public spaces to collect unconstrained environment data. In this paper, we use the test data from steps 1, 2, 3, 5, and 6 in our analysis. The test data from step 4 is not analyzed in this paper. During step 4, the participant repeated the same course four times with the phone reoriented in a different position each time. We discuss the issue of how the phone is carried in Section 4.

Each participant was filmed throughout the experiment using a handheld camera. The video footage was used to manually annotate the sensor data with ground truth activity labels. The accelerometer and gyroscope measurements from the smartphone, along with the annotated activity labels from video footage, are publicly available on Zenodo [26].

### 2.2. The Movelet Method: Single Sensor

The movelet method, proposed by Bai et al. [24], was originally designed for activity recognition from a body-worn tri-axial accelerometer, but it can be applied to any single tri-axial sensor. In our previous paper, we applied the movelet method separately to smartphone accelerometer data and smartphone gyroscope data [11]. The method proposed by Bai et al. [24] has the following procedure.

The movelet method uses pattern recognition or pattern matching. Consider a single tri-axial sensor (e.g., a smartphone accelerometer). For a given participant, let S(t)=(x(t),y(t),z(t)) denote the vector of *x*, *y*, and *z* measurements taken by the sensor at time *t* for the participant. We will assume that the sampling frequency of the sensor is 10 Hz. A movelet is defined as a 1-s window of the sensor’s data. Let M(t) denote a movelet beginning at time *t*. Then we have

(1) M(t)={S(t),S(t+0.1),S(t+0.2),...,S(t+0.9)},

where the time *t* is in units of seconds. Thus, the movelet M consists of the time series from all three axes (*x*,*y*,*z*) of the sensor within a second. The time increments are spaced by 0.1 s because this is the reciprocal of the sampling frequency 10 Hz. We set the movelet duration to be 1-s long based on existing literature. Bai et al. [24] found 1 s to be an appropriate choice because a 1-s window strikes a balance between being long enough to differentiate activities, yet short enough to avoid encapsulating multiple activities.

Before applying the movelet method, a dictionary is constructed from a participant’s training data. In the dictionary, the movelets derived from the training smartphone data are grouped into categories of different activities, based on the ground truth activity labels. In applying the movelet method, new movelets are constructed from new smartphone data. Each new movelet is compared to the movelets in the dictionary, and the most similar dictionary movelet is used to classify its activity. To distinguish between dictionary movelets and new movelets, define T to be the set of times *t* during training data collection, and V to be the set of times *t* during new data collection. Any given dictionary movelet M(t) has time t∈T, while any given new movelet M(t) has time t∈V. The process of obtaining and comparing dictionary movelets and new movelets is described in the following paragraphs.

First, the study investigator makes a comprehensive list of daily life activities. Let *A* denote the number of activities in the list, which consists of activity 1, activity 2, up through activity *A* (e.g., walk, sit, stand). Training data are gathered by having the participant perform each of the activities while collecting data from the sensor of interest (e.g., a smartphone accelerometer). These training data are then used to build the dictionary for this participant. Each of the *A* activity entries in the dictionary are composed of multiple movelets, where any given movelet is a 1-s window of the sensor’s tri-axial (*x*,*y*,*z*) data. For each of the *A* activity entries, the collection of dictionary movelets is obtained using a sliding window process, as described in the following example. Suppose we have a 5-s segment of training data for a given activity. Then one obtains the 1-s dictionary movelets for the activity entry by sliding a 1-s window forward one sample (0.1 s, the reciprocal of sampling frequency) at a time along the tri-axial data, until the right end of the 1-s window meets the last point of the 5-s time series. The resulting number of movelets is 41 from the 5-s time series. The number of dictionary movelets of an entry depends on the data collection frequency and the duration of the training data for the activity. In summary, every dictionary movelet M(t) (t∈T) is linked to a particular activity entry L(t) in the list of *A* activities.

Next, we perform activity classifications on new data (termed test data here) using the dictionary. For the test data, we also construct movelets by sliding a 1-s window forward, one sample (0.1 s) at a time along the test data time series. Each test movelet is then matched with one of the dictionary movelets based on the smallest discrepancy. Precisely, for a given test movelet $M(t_{0})$ ($t_{0}\in V$), we find

(2) $$t^{*}=\arg\;\min_{t\in T}D_{S}\left(M\left(t\right),M\left(t_{0}\right)\right).$$

The function $D_{S}$ is a discrepancy metric using Euclidean distance that will be defined in Section 2.3 [24]. Intuitively, Equation (2) finds the dictionary movelet $M(t^{*})$ with the lowest discrepancy from the test movelet $M(t_{0})$. The activity label $L(t^{*})$ of the dictionary movelet $M(t^{*})$ is then assigned to the test movelet $M(t_{0})$ as its classification. To classify the activity at a given time point *t*, one uses the test movelet beginning at the time point *t* and the subsequent nine following it. A majority vote is taken among these movelets, where the activity that receives the most votes is taken as the classification for the time point *t*. The rationale behind the majority vote process is that later movelets also contain activity information for the time point because human activities are continuous.

An advantage of the movelet method is its small training data requirement. As demonstrated by Bai et al. [24], only a few seconds of training data are required per activity. More sophisticated machine learning methods can be applied to link the windows of data to activities [12,18], but this requires more training data [24].

### 2.3. Discrepancy Metric

In the single-sensor method, a discrepancy metric called $D_{S}$ is used to compare each test movelet to each dictionary movelet [24]. The discrepancy $D_{S}$ is defined as follows. Consider a dictionary movelet M(t) and test movelet $M(t^{\prime })$. For simplicity, we remove the *t* and $t^{\prime }$ and refer to these movelets as M and $M^{\prime }$, respectively. For the dictionary movelet M, here we let X be the vector of length *n* containing the time series data for the *x*-axis of the single sensor during the 1-s window. Define the vectors Y and Z analogously. To be precise, we have $X=\left(x_{1},x_{2},\ldots ,x_{n}\right)$, $Y=\left(y_{1},y_{2},\ldots ,y_{n}\right)$, and $Z=\left(z_{1},z_{2},\ldots ,z_{n}\right)$, where n=10. The subscripts 1 through *n* represent the different times in the 1-s window. For the new movelet $M^{\prime }$, we analogously define $X^{\prime }=\left(x_{1}^{\prime },x_{2}^{\prime },\ldots ,x_{n}^{\prime }\right)$, $Y^{\prime }=\left(y_{1}^{\prime },y_{2}^{\prime },\ldots ,y_{n}^{\prime }\right)$, and $Z^{\prime }=\left(z_{1}^{\prime },z_{2}^{\prime },\ldots ,z_{n}^{\prime }\right)$. Using A and $A^{\prime }$ to represent a pair of vectors of a given dimension from the dictionary movelet M and test movelet $M^{\prime }$, respectively, the Euclidean distance for the dimension is defined as

$$d_{L_{2}}\left(A,A^{\prime }\right)=\sqrt{\sum_{i=1}^{n}{\left(a_{i}-a_{i}^{\prime }\right)}^{2}}.$$

In the discrepancy metric, the Euclidean distance is computed for each of the *x*, *y*, and *z* axes, and these three distances are averaged together. Thus, the discrepancy $D_{S}$ between the two movelets M and $M^{\prime }$ is:

$$D_{S}\left(M,M^{\prime }\right)=\frac{1}{3}\left[d_{L_{2}}\left(X,X^{\prime }\right)+d_{L_{2}}\left(Y,Y^{\prime }\right)+d_{L_{2}}\left(Z,Z^{\prime }\right)\right].$$

### 2.4. The Movelet Method: Joint Sensors

In this paper, we propose an extension to the movelet method in which we use gyroscope and accelerometer data simultaneously. The motivation behind this is that we hypothesized that acceleration and angular velocity provide different physical information, and combining both could improve the accuracy of the activity classifications. Related work includes He et al. [25], who applied the movelet method with data from multiple accelerometers fixed to different parts of the body. The joint-sensor method using accelerometer and gyroscope data follows the same procedure as in Section 2.2 and Section 2.3, except for the key differences that are described below in this section.

In the joint-sensor method, the 1-s movelets include all six dimensions of data (*x*, *y*, *z* from the accelerometer and gyroscope sensors) rather than only three dimensions (from a single sensor). Thus, these movelets are multistream movelets because they come from the accelerometer and gyroscope data streams. The multistream movelets still follow Equation (1), except the data S(t) at any given time *t* is now a vector of six values, with

$$S\left(t\right)=(x_{a}\left(t\right),y_{a}\left(t\right),z_{a}\left(t\right),x_{g}\left(t\right),y_{g}\left(t\right),z_{g}\left(t\right)),$$

where $x_{a}$, $y_{a}$, and $z_{a}$ correspond to the accelerometer and $x_{g}$, $y_{g}$, and $z_{g}$ correspond to the gyroscope. In our data collection, the sampling frequencies for the accelerometer and gyroscope were both 10 Hz. However, the measurements from the two sensors were not synchronized. Thus, we required data preprocessing to synchronize the accelerometer and gyroscope data before implementing the joint-sensor method. In our data preprocessing, we linearly interpolated the gyroscope data to the timestamps in the accelerometer data. This was done for both training data and test data. Thus, all dictionary and test movelets in the joint-sensor analysis had six measurements at every accelerometer timestamp. We chose linear interpolation because it is simple and has been recommended for this type of data [27].

Compared to the single-sensor method, we also used a different discrepancy metric called $D_{J}$ in the joint-sensor method to compare our multistream movelets. The discrepancy metric $D_{J}$ is defined as follows. As in Section 2.3, we use M and $M^{\prime }$ to represent a dictionary movelet and test movelet, respectively. Note that now the dictionary movelet M has corresponding data vectors $X_{a}$, $Y_{a}$, and $Z_{a}$ for the accelerometer and vectors $X_{g}$, $Y_{g}$, and $Z_{g}$ for the gyroscope. Analogously, the test movelet $M^{\prime }$ has corresponding data vectors $X_{a}^{\prime }$, $Y_{a}^{\prime }$, and $Z_{a}^{\prime }$ for the accelerometer and vectors $X_{g}^{\prime }$, $Y_{g}^{\prime }$, and $Z_{g}^{\prime }$ for the gyroscope. Moreover, as described above, the vectors for the gyroscope (i.e., $X_{g}$, $Y_{g}$, and $Z_{g}$ for movelet M and $X_{g}^{\prime }$, $Y_{g}^{\prime }$, and $Z_{g}^{\prime }$ for movelet $M^{\prime }$) are obtained by interpolating the original gyroscope data to the accelerometer timestamps, so that the two data sources are synchronized.

The distance metric $D_{j}$ for comparing M and $M^{\prime }$ is defined as:

$$\begin{array}{c} D_{J}\left(M,M^{\prime }\right)=\frac{1}{6}[d_{L_{2}}\left(X_{a},X_{a}^{\prime }\right)+d_{L_{2}}\left(Y_{a},Y_{a}^{\prime }\right)+d_{L_{2}}\left(Z_{a},Z_{a}^{\prime }\right)\\ +d_{L_{2}}\left(X_{g},X_{g}^{\prime }\right)+d_{L_{2}}\left(Y_{g},Y_{g}^{\prime }\right)+d_{L_{2}}\left(Z_{g},Z_{g}^{\prime }\right)]. \end{array}$$

Thus, for the joint-sensor movelet method, we compute the distances for the *x*, *y*, and *z* axes of both the accelerometer and gyroscope and average these six distances together.

### 2.5. Analysis Procedure

We applied the extended version of the movelet method using accelerometer and gyroscope data jointly, and we also compared its classification accuracy to the original (i.e., single-sensor) movelet method using the accelerometer data only and the gyroscope data only. Table 1 summarizes the key points of the analysis procedure. In the gyroscope-only analyses, we used the original gyroscope data rather than interpolated gyroscope data. This was done to mimic how an analysis using only gyroscope data would be performed in practice. Applying the movelet method to the original gyroscope data resulted in an activity classification for each gyroscope timestamp. Since the accelerometer-only and joint-sensor analyses yielded classifications at the accelerometer timestamps, we then computed activity classifications for each accelerometer timestamp by taking the classification for the closest gyroscope timestamp. The R code for this paper is provided on GitHub at https://github.com/KebinYan/Code-for-Paper; accessed on 24 February 2022. The analyses were performed using a MacBook Pro laptop with a dual-core Intel Core i5 processor running at 2.7 GHz and 8 GB of 1967 MHz DDR3 onboard memory.

## 3. Results

### 3.1. Training Data Example

**Figure 1:**
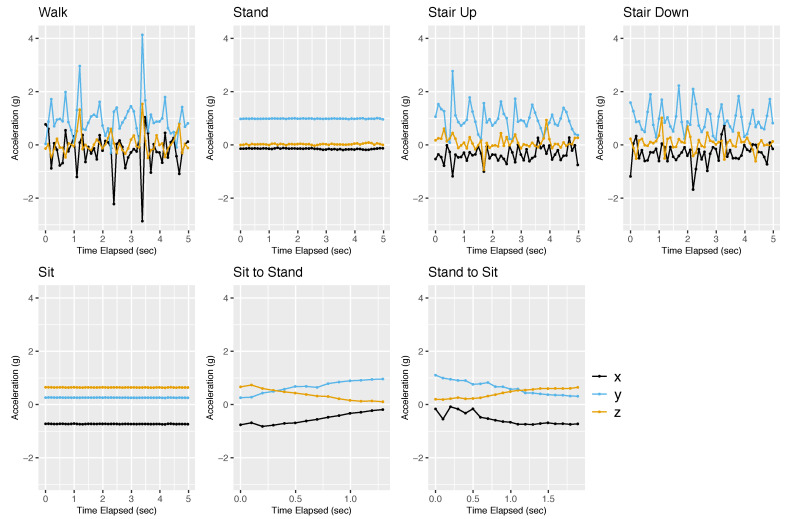
Accelerometer training data for participant 1. This figure shows the accelerometer training data for participant 1. The accelerometer measures the acceleration of the smartphone along the *x*, *y*, and *z* axes. The acceleration data are measured in units of *g* (i.e., 9.81 m/s${}^{2}$). The *x*-axis is shown in black, the *y*-axis in blue, and the *z*-axis in orange.

**Figure 2:**
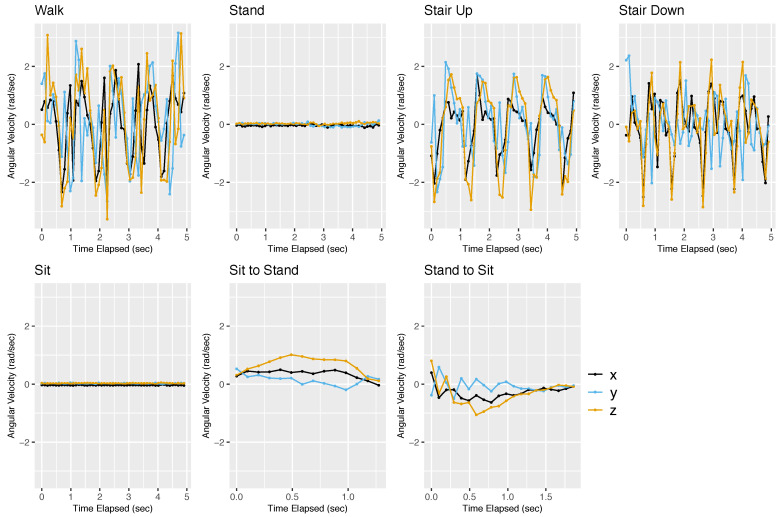
Gyroscope training data for participant 1. This figure shows the gyroscope training data for participant 1. The gyroscope measures the angular velocity of the smartphone projected onto the *x*, *y*, and *z* axes. The angular velocity data are measured in units of radians per second (rad/s). The *x*-axis is shown in black, the *y*-axis in blue, and the *z*-axis in orange.

We built a separate dictionary for each participant using her/his training data, with up to 5 s per activity. As an example, Figure 1 and Figure 2 show the training data of participant 1 from the accelerometer and gyroscope, respectively. For both sensors, the signals for standing and sitting are flat lines, while those for walking, ascending stairs, and descending stairs are variable and quasi-periodic. The signals for the sit-to-stand and stand-to-sit activities are smooth curves that occur over a brief period of time.

In the accelerometer training data (Figure 1), we observe that each activity has its own characteristic signature. For example, standing and sitting can be distinguished since the *x*, *y*, and *z* data are at different levels. In standing, *y* is approximately +0.98 g and *x* and *z* are close to −0.15 g and 0.02 g, respectively, likely corresponding to the phone being positioned vertically. In sitting, *x* is approximately −0.73 g, *z* is approximately 0.64 g, and *y* is approximately 0.26 g. This may correspond to the phone being in the right pocket. The curves for stand-to-sit are a smooth transition from the coordinates for standing to sitting, and vice versa for sit-to-stand. The patterns for walking, ascending stairs, and descending stairs differ from each other (e.g., the shapes in the *z*-axis data are different). Moreover, within an activity, the three axes also show different signatures (e.g., different fluctuations and peaks). For example, in walking, the *x* and *y* have an out-of-phase tendency in their extremes but the *z* shows more degrees of freedom.

In the gyroscope training data (Figure 2), we also observe characteristic signatures for different activities. These signatures are different from what we saw in the accelerometer data. In both standing and sitting, the *x*, *y*, and *z* are all approximately 0 radians/s because the phone is not rotating. Thus, given a short segment of the gyroscope signal, we may not be able to distinguish sitting from standing. However, the transitions sit-to-stand and stand-to-sit can be distinguished by the gyroscope. The sit-to-stand transition shows smooth arcs that start and end at 0. In contrast, the stand-to-sit transition is more variable in the beginning and then approaches 0 smoothly. For the activities of walking, ascending stairs, and descending stairs, the patterns appear to be more periodic for the gyroscope than for the accelerometer. The three axes are also more synchronized. Therefore, the accelerometer and gyroscope data are complementary to each other.

### 3.2. Results for Participant 3

We applied the movelet method to steps 1, 2, 3, 5, and 6 of each participant’s test data. Table 2 shows the amount of test data per participant. In this section, we present the joint-sensor results of participant 3 as an example. We also compare them to the accelerometer-only and gyroscope-only results. In Section 3.3, we then summarize the joint-sensor results of all participants.

**Figure 3:**
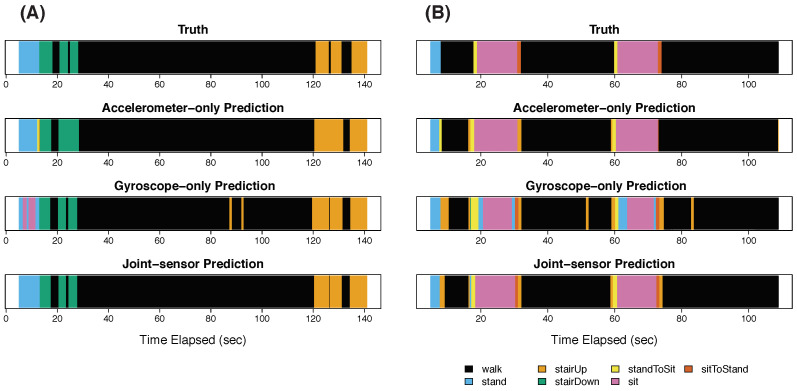
Steps 1 and 2 for participant 3. Panels (**A**,**B**) show the results for participant 3 in steps 1 and 2, respectively. For each panel, four figures are shown. The top figure gives the true activity labels based on video footage. The second figure shows the activity classifications from the accelerometer-only method, the third figure shows the classifications from the gyroscope-only method, and the fourth shows the classifications from the joint-sensor method. In each row, the horizontal axis gives the time elapsed, measured in seconds.

Figure 3A shows the results for participant 3 in step 1. In step 1, the participant performed the activities of standing (blue), descending stairs (green), walking (black), and ascending stairs (orange). The top image in Figure 3A shows the true activity labels based on video footage, the second image shows the classifications for accelerometer-only, the third shows the classifications for gyroscope-only, and the fourth shows the classifications for the joint-sensor method.

Both the accelerometer-only and gyroscope-only methods recognized descending stairs and ascending stairs accurately. The accelerometer-only method also correctly recognized standing. However, the gyroscope-only method misclassified 47.5% of the standing period as sitting (pink). This is consistent with the visual examination of the training data that sitting and standing periods are not distinguishable from gyroscope data. For the walking (black) period, the accelerometer-only method matched the truth accurately. While the gyroscope-only classifications were also mostly accurate, there were two segments that were characterized as ascending stairs. Further examination is needed as to whether this is an artifact or reflects small changes on the ground.

Combining the accelerometer and gyroscope data, the accurate ascending stair and descending stair classifications were preserved. Moreover, standing was classified correctly, improved from the gyroscope-only classifications. The walking classifications were also accurate. In particular, the joint-sensor method corrected the two segments misclassified as ascending stairs from the gyroscope alone. The joint-sensor method also captured the brief periods of walking that fell between ascending or descending stairs, which were sometimes smoothed over by the accelerometer alone.

Figure 3B shows the results for step 2. During this step, the participant performed the activities of standing (blue), walking (black), stand-to-sit (yellow), sitting (pink), and sit-to-stand (red). The joint-sensor method and accelerometer-only method classified the sitting periods (pink) accurately. The gyroscope-only method characterized these periods correctly most of the time, but misclassified the start and end of each sitting period as standing. The walking periods were classified well by all three methods, with the joint-sensor and accelerometer-only performing the best. In step 2, the joint-sensor method had improved accuracy for walking compared to the gyroscope-only method, correcting the two false positives of ascending stairs that occurred during the second and third walking periods. Compared to gyroscope alone, the joint-sensor method also reduced the length of the ascending stair error at the beginning of the first walking period. All three methods picked up the two stand-to-sit transitions (yellow). However, the gyroscope-only method overestimated the length of the first stand-to-sit transition. The joint-sensor method reduced the length closer to the truth. The sit-to-stand transitions (red) were almost entirely missed by the accelerometer-only method, but they were captured by the joint-sensor and gyroscope-only methods.

**Figure 4:**
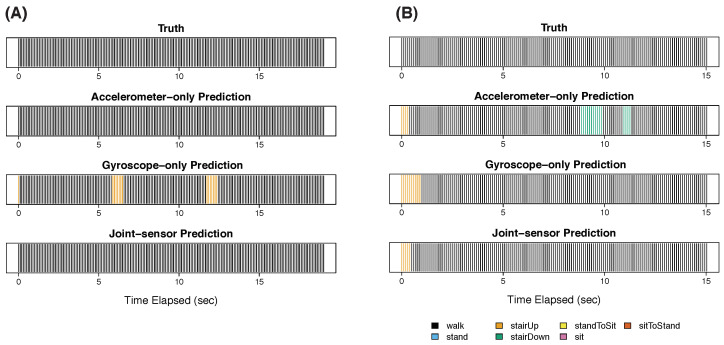
Step 3 for participant 3: normal and fast walking. This figure presents the results of participant 3 during step 3 for normal-paced walking Panel (**A**) and fast-paced walking Panel (**B**).

**Figure 5:**
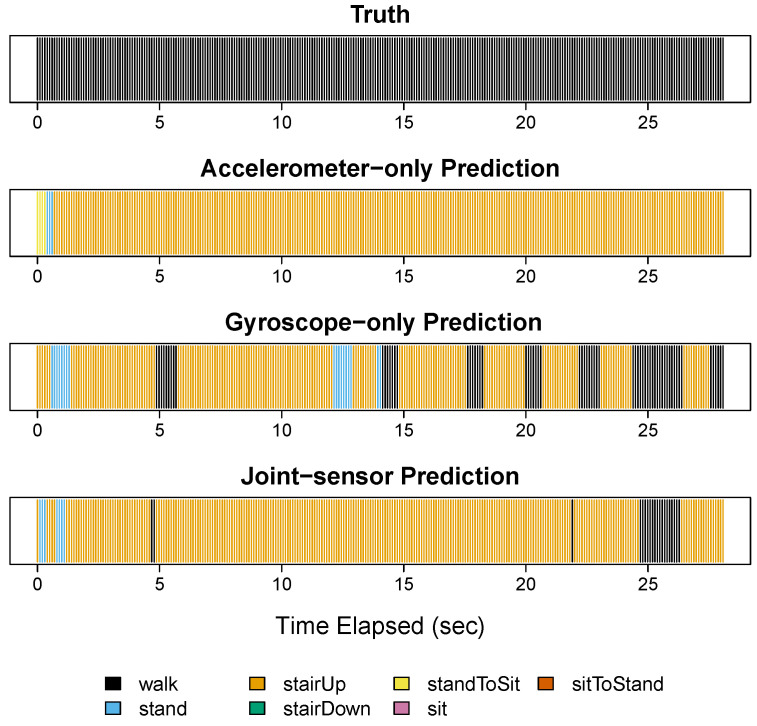
Step 3 for participant 3: slow walking. This figure presents the results of participant 3 during step 3 for slow walking.

In step 3, the participant walked at three different speeds: normal (Figure 4A), fast (Figure 4B), and slow (Figure 5). All three methods correctly classified the normal walk (Figure 4A) for most (or all) of the time. The gyroscope-only method had two false positives of ascending stairs, which were corrected by the joint-sensor method. For fast walking (Figure 4B), all three methods were accurate for most of the time, with the joint-sensor method performing the best. The gyroscope-only method incorrectly predicted a 1-s episode of ascending stairs at the beginning of the fast walk. The joint-sensor method shortened this error by about one half. The accelerometer-only method also predicted an episode of ascending stairs at the beginning, as well as two episodes of descending stairs. The joint-sensor method was able to eliminate the two erroneous episodes of descending stairs, though the ascending stair error remained. All three methods showed large errors in classifying slow walking (Figure 5). The accelerometer-only method misclassified the entire slow walking period, using ascending stairs for most of the time. The gyroscope-only method also chose ascending stairs for most of the slow walking period, though it captured some episodes of walking. The joint-sensor method performed better than the accelerometer only by classifying some brief periods as walking, but it performed worse than the gyroscope alone. One might expect that the classification becomes harder when the pace of walking is slower because the slower pace can match the pace of movement of other activities, such as ascending stairs. The three methods also had difficulty in classifying slow walking for the other participants of the study. We discuss this further in Section 4.

**Figure 6:**
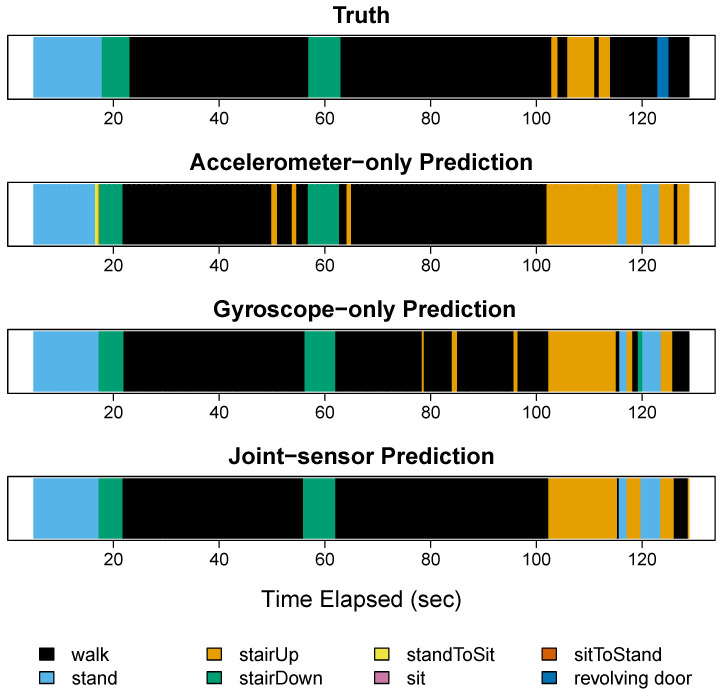
Step 5 for participant 3. This figure presents the results for participant 3 during step 5. In step 5, the participant performed the activities of standing, descending stairs, walking, ascending stairs, and going through a revolving door. The revolving door activity is not included in the participant’s dictionary.

In step 5 (Figure 6), the participant performed the activities of standing (blue), descending stairs (green), walking (black), ascending stairs (orange), and going through a revolving door (dark blue). All three methods accurately classified the two periods of descending stairs. They also performed well at classifying the two extended walking periods, with the joint-sensor method performing the best. During these walking periods, the accelerometer-only method and gyroscope-only method each sometimes confused walking for bursts of ascending stairs. These errors were corrected by the joint-sensor method. All three methods struggled at the end of step 5 when the participant went through the revolving door (dark blue). This is because going through a revolving door was not in the dictionary, so all methods used other activities to substitute it, including standing and ascending stairs. There was a walking (black) period directly before the participant went through the revolving door. The three methods misclassified most of this walking period using standing and ascending stairs. This may be due to the participant’s reduction in walking speed before entering the revolving door.

**Figure 7:**
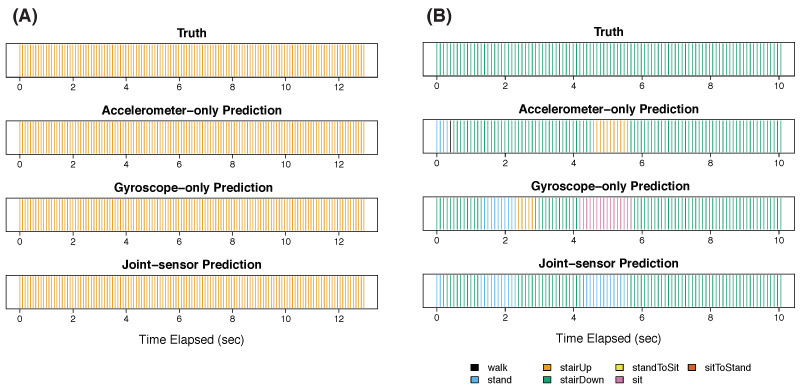
Step 6 for participant 3. This figure presents the results for participant 3 during step 6. In step 6, the participant ascended and descended a staircase. Panel (**A**) corresponds to ascending stairs and Panel (**B**) to descending stairs.

In step 6 (Figure 7), the participant ascended (Figure 7A) and descended (Figure 7B) a staircase. All three methods correctly classified ascending stairs, as shown in Figure 7A. The methods also correctly classified descending stairs (Figure 7B) for most of the time, with the accelerometer-only performing the best. The descending stair period in step 6 was correctly classified for about 84% of the time using the accelerometer alone, 70% using the gyroscope alone, and 75% using the sensors jointly. The accelerometer-only method confused the beginning of the period with standing, and the middle portion with ascending stairs. The gyroscope-only method had more errors, including episodes of standing, ascending stairs, and sitting. Combining the sensors improved the results from the gyroscope alone, eliminating the ascending stair error, as well as shortening the length of the sitting error and changing it to standing, which is closer to the ground truth activity of descending stairs. Combining the sensors reduced the length of the standing error from the accelerometer-only method. However, it also added a new standing error at about 1.3 s elapsed, originating from the gyroscope data.

**Figure 8:**
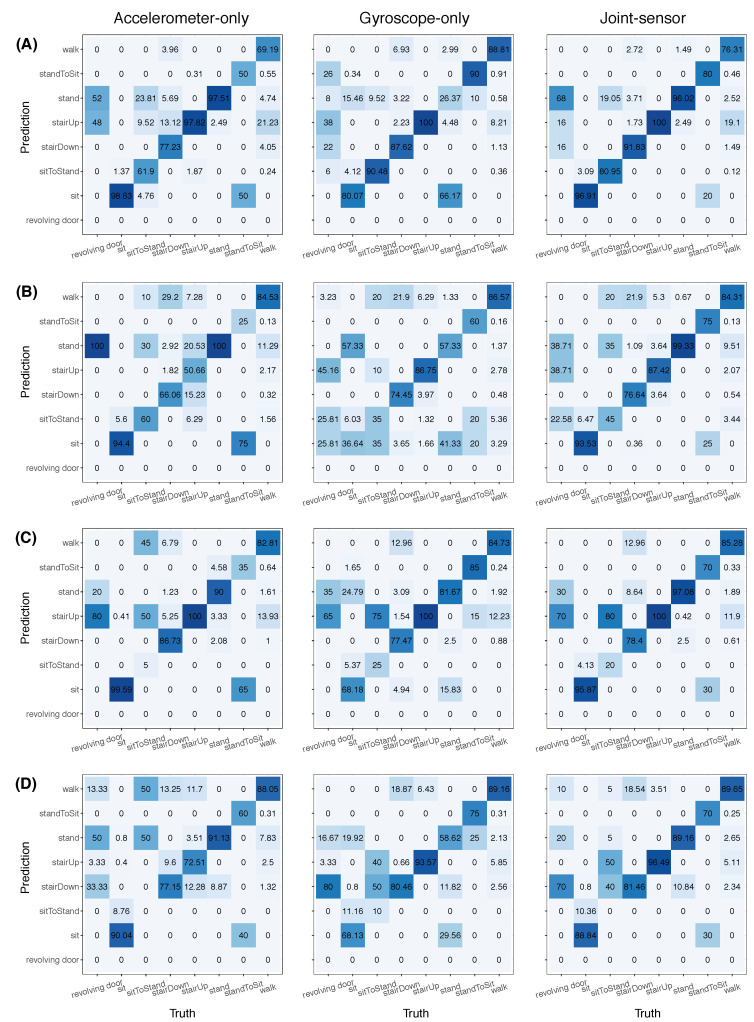
Confusion matrices for accelerometer-only, gyroscope-only, and joint-sensor methods. This figure presents the confusion matrices for each participant. Row (**A**) corresponds to participant 1, row (**B**) to participant 2, row (**C**) to participant 3, and row (**D**) to participant 4. For each participant, there are three confusion matrices corresponding to accelerometer-only (column 1), gyroscope-only (column 2), and joint-sensor (column 3). These confusion matrices incorporate steps 1, 2, 3, 5, and 6 in test data collection. In each confusion matrix, the ground truth activity labels are on the bottom margin, and the predicted activity labels are on the left margin. Each column shows the distribution of the predicted labels for the corresponding ground truth activity label. Thus, every column sums to 100%.

The results of participant 3 from steps 1, 2, 3, 5, and 6 are quantified in row C of Figure 8. The figure includes confusion matrices for the accelerometer-only (column 1), gyroscope-only (column 2), and joint-sensor (column 3) methods. Each row corresponds to a different participant, with participant 3 in row C. In each confusion matrix in Figure 8, any given column corresponds to a unique ground truth activity label, and the values in the column show the distribution of the predicted activity labels for the given ground truth activity label. We measure accuracy by the diagonal elements of the confusion matrices.

The results for participant 3 in Figure 8 are consistent with our observations from Figure 3, Figure 4, Figure 5, Figure 6 and Figure 7. For example, the gyroscope-only method (row C, column 2) tended to confuse standing and sitting, classifying 25% of the “sit” labels as “stand” and 16% of the “stand” labels as “sit”. In contrast, the joint-sensor method (row C, column 3) classified standing correctly 97% of the time and sitting correctly 96% of the time. The accelerometer-only method (row C, column 1) had high accuracies for most of the activities (i.e., dark shading in the diagonal entries), but classified the “sit-to-stand” labels with only 5% accuracy. All three methods classified the ascending stair (“stairUp”) labels correctly. The accelerometer-only method had higher accuracy than the gyroscope-only method for standing, sitting, and descending stairs. The gyroscope-only method had higher accuracy than the accelerometer-only method for walking, sit-to-stand, and stand-to-sit. For all activities, the accuracy value of the joint-sensor method was either between that of the accelerometer-only and gyroscope-only methods, or was higher than both of them.

### 3.3. Quantifying Classification Accuracy

**Figure 9:**
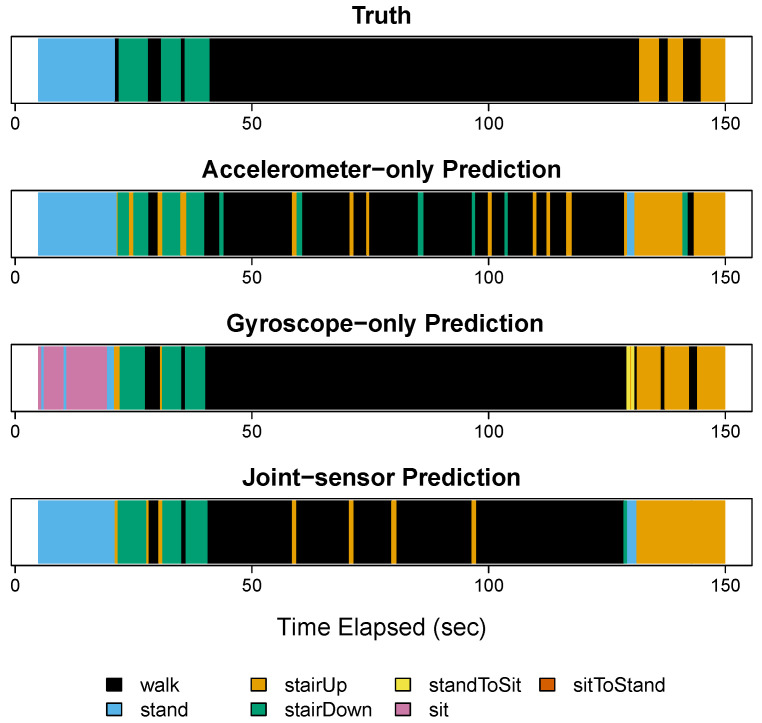
Step 1 for participant 1. The figure shows the results for participant 1 during step 1.

In this section, we discuss the results for the other participants. The confusion matrices for participants 1, 2, and 4 are shown in rows A, B, and D, respectively, of Figure 8. Moreover, the counterparts to Figure 3, Figure 4, Figure 5, Figure 6 and Figure 7 for these other participants are shown in the Supplement (Figures S1–S8). There are similarities across the participants. For instance, as with participant 3, the gyroscope-only method tended to mix up standing and sitting for all participants. The joint-sensor method was able to correct most of these errors. We also observed that the joint-sensor method could correct systematic errors from one sensor in misidentifying walking as another activity. In participant 3, the classifications of the gyroscope-only method sometimes misrepresented walking as short bursts of ascending stairs, as discussed in Section 3.2. The joint-sensor method was able to correct many of these errors. This same pattern of correction also occurred for participants 1 and 4. We should note that the systematic errors were not exclusive to one particular sensor. An interesting difference between the participants was that, while the short errors mostly occurred from gyroscope-only for participant 3, they tended to be from the accelerometer-only for participants 1 and 4. As an example, we show the step 1 result for participant 1 in Figure 9. For walking (black), the accelerometer-only method characterized many short segments as ascending stairs or descending stairs. The joint-sensor method reduced the number of these false positives that occurred in the classifications from the accelerometer alone. We believe the reason that the joint-sensor method could generate improvement was that it was rare for both sensors to misidentify walking at the same time with the same activity. The error might occur in accelerometer only or gyroscope only, but not both.

The joint-sensor method did not always achieve superior results over a single-sensor method. This was particularly the case for participant 1. For instance, in Figure 9 (participant 1, step 1), the joint-sensor method slightly degraded the walking classifications by the gyroscope only. This is because some false positives of ascending stairs originating from the accelerometer data remained in the joint-sensor classifications. We also found that some short periods of walking between ascending stairs that were picked up by the gyroscope were smoothed out by the joint-sensor method. On the other hand, the joint-sensor method still has advantages over using a single sensor. This is because “which” single-sensor method (accelerometer or gyroscope) performed better was not consistent across participants. In such situations, the joint-sensor method generally provided a closer result to the better performing single-sensor method for the given participant.

Table 3 summarizes the results from Figure 8 by looking at the activities in groups. Four activity groups were considered, including (A) all activities, (B) vigorous activities, (C) stationary activities, and (D) transition activities. The table presents the average accuracy value of each method for each participant in each activity group. We first focus on Section A of Table 3, which corresponds to the all activities group. This group consists of walking, standing, ascending stairs, descending stairs, sitting, the sit-to-stand transition, and the stand-to-sit transition. Thus, the average accuracy in Section A was computed by averaging all of the diagonal elements of the corresponding confusion matrix, except for “revolving door”. The revolving door activity was excluded since it was not part of the participants’ dictionaries. For the all activities group, the accelerometer-only and gyroscope-only columns are similar to each other. This means that, for each participant, the average accuracy value for the accelerometer-only method was close to that of the gyroscope-only method. The average accuracy tended to improve after combining the accelerometer and gyroscope data. The degree of improvement varied by participant. The percent increase in average accuracy from the joint-sensor method (relative to the higher average accuracy value between accelerometer-only and gyroscope-only) was 10.4% for participant 1, 16.7% for participant 2, 4.7% for participant 3, and 7.7% for participant 4.

To assess which activities yielded the largest improvements, we divided the activities into smaller groups, including vigorous activities, stationary activities, and transition activities. The vigorous activities included walking, ascending stairs, and descending stairs. The stationary activities included standing and sitting. Lastly, the transition activities included stand-to-sit and sit-to-stand. We present the average accuracy values for each of these activity groups in Table 3, with vigorous activities in Section B, stationary activities in Section C, and transition activities in Section D.

For the vigorous activities (Table 3, Section B), the joint-sensor method had higher average accuracy than the accelerometer-only method for all participants, except for participant 3. The percent improvement in average accuracy was 9.8% for participant 1, 23.4% for participant 2, and 12.6% for participant 4. For participant 3, there was a decrease in average accuracy of 2.1%. For all participants, the average accuracy of the joint-sensor method was close to that of the gyroscope-only method. Relative to the gyroscope-only method, there was a 2.9% decrease in average accuracy for participant 1, a 0.24% increase for participant 2, a 0.57% increase for participant 3, and a 1.7% increase for participant 4. Thus, for the vigorous activities, the improvement of the joint-sensor method was mostly from correcting the errors of the accelerometer-only method.

For the stationary activities (Table 3, Section C), the average accuracy for the joint-sensor method was considerably higher than for the gyroscope-only method. This was because the mix-up between standing and sitting was corrected by including accelerometer data. For example, the average accuracy of participant 2 was 97.2% for accelerometer-only, 47.0% for gyroscope-only, and 96.4% for the joint-sensor method. Hence, for the stationary activities, the improvement of the joint-sensor method was mainly from correcting the errors of the gyroscope-only method.

For the transition activities (Table 3, Section D), the average accuracy values of the joint-sensor method tended to be low for most participants. When used on their own, the sensors each had difficulty recognizing the transition activities as well. The low accuracy rates may partly be due to the movelet method. For example, although the method might detect the transition activities, the classifications may be shifted slightly too soon in time. This is because the classification at any given time point involves taking a majority vote among the movelet beginning at the time point and the subsequent nine following it. Since the transition activities are momentary, some movelets could be picking up the activity occurring after the transition.

Overall, we find that the different sensors play different roles in correcting classification errors. The joint sensor method can correct the shortcomings of the gyroscope-only method in standing and sitting. It can also correct the shortcomings from the accelerometer-only method in the vigorous activities. These findings are consistent with the physical nature of the sensors.

## 4. Discussion

This study found that combining accelerometer and gyroscope data can result in more accurate activity recognition. For example, the gyroscope-only method had difficulty in differentiating between the activities of standing and sitting, but combining the accelerometer with gyroscope data largely corrected this error. For the activity of walking, combining the accelerometer and gyroscope data improved the accuracy compared to the accelerometer alone in some cases (e.g., participant 1) and to the gyroscope alone in other cases (e.g., participant 3). Although the single-sensor methods using the accelerometer or gyroscope classified ascending and descending stairs to a certain degree, the combined method using both sensors made further improvements in some cases. Our results also showed that for certain types of movement, a properly chosen single-sensor method may be adequate, e.g., accelerometer for stationary activities. These findings highlight the close connections among the specification of scientific questions (e.g., what activities are of interest?), the choice of data types (whether to collect accelerometer data, gyroscope data, or both), and the choice of the data analysis method.

This work expands the range of usage of the movelet method. Previous work on the movelet method mostly concentrated on using a single body-worn accelerometer, or multiple accelerometers fixed to different parts of the body. We extended the movelet method to incorporate different types of sensors from one smartphone. This takes full advantage of the smartphone as a compact and convenient to carry all-in-one instrument, which can sense different types of movement simultaneously over long time periods.

The movelet method is a useful classification tool that is simple to implement in research and clinical settings. Our analyses showed that the movelet method is fairly accurate. Compared with other statistical methods, this relatively simple approach has the advantage of being transparent, intuitive, and interpretable. Therefore, the movelet method can be used together with more complex methods, such as deep learning, so that we can gain more insight into the classification procedure. Moreover, given that the movelet method makes activity classifications for each person based on her/his own dictionary, the classifications are personalized to the individual’s unique data patterns and, therefore, account for factors, such as the person’s height, weight, age, and health conditions. Models built using training data from one cohort (e.g., young, healthy people) may perform poorly when applied to another group (e.g., older adults or patients with illnesses) [28,29].

As our analysis results show, one remaining problem is that the joint-sensor method and the two single-sensor methods all had difficulty with accurately classifying slow walking. To address this issue in future work, we plan to develop an extension to the joint-sensor method that allows for movelet transforms, which stretch or compress the 1-s dictionary movelets [30]. The purpose of movelet transforms is to improve activity recognition in cases where the participant performs a given activity at a different pace during testing, compared to during training. These transforms may be helpful in improving the accuracy of slow walking recognition. They can also be adaptable in cases where a patient’s condition evolves over time (e.g., a patient’s walking pace may increase over time as she/he recovers from surgery). In future work, we also plan to use smartphone sensor data to examine a patient’s gait patterns, in addition to performing her/his activity recognition. For example, there is existing work about analyzing the human gait using sensor data, including estimating gait parameters (e.g., average stride duration) as well as detecting abnormalities in gait [31,32]. In future studies, we can investigate methods to analyze the human gait using smartphone accelerometer and gyroscope data jointly.

One limitation of this work is that we studied the specific case where the phone is worn in the pocket. In reality, the phone can be carried in different locations (e.g., pocket, hand, backpack, purse) that can change with time. The specific context may also differ (e.g., phone in a tighter pocket or oriented in a different direction). An area of future work is to extend the joint-sensor method to accommodate these changes robustly. One approach is to identify the location of the smartphone placement based on the accelerometer and gyroscope data, and then apply the appropriate dictionary accordingly. We may also consider standardizing the training and testing data based on the phone’s placement to reduce the context influence on the amplitude.

This work was a pilot study using a small sample size collected by the investigators. The small sample size is a limitation of this study. Our goal in this pilot study was to understand each sensor’s role and how the combination of the sensors could provide further information. To achieve this goal, we performed a detailed analysis at the highest possible frequency, verifying the activity classification at each time point and for each activity. We believe these results can apply in more general situations, but this should be confirmed in a study with a larger sample size. We are planning such a data collection.

In our future work, we will further develop the movelet method and apply it in free-living environments. On the one hand, we will thoroughly evaluate the performance measures of the joint-sensor method, including sensitivity, specificity, and precision for each activity type. This is aligned with our current planning for a major effort to collect multi-sensor data with a large sample size of diverse participants. On the other hand, we will improve the movelet methodology and combine it with other advanced statistical tools. First, we will build more sophisticated dictionaries with more categories of activities. Future data collection and analyses can incorporate new activities that are not in the current dictionaries, such as the activity of running. Second, we will automate the customized dictionary generation process for each individual person using machine learning techniques. In addition to these major developments, we will fine-tune and expand the joint-sensor method that we are using now. For example, our current analysis applied linear interpolation to interpolate the gyroscope data to the accelerometer timestamps. An area of future work is to test other interpolation methods, such as cubic splines or B-splines. Moreover, based on our analyses, gyroscope and accelerometer data seemed to play different roles in identifying different types of movement. To take advantage of these differences, we will evaluate whether assigning different weights to the two sensors and their axes (*x*, *y*, and *z*) can improve the accuracy of the joint-sensor method.

## Figures and Tables

**Table 1:** Analysis procedure. This table summarizes the analysis procedure of this paper. The rows of the table show the methods, including the joint-sensor method, the accelerometer-only method, and the gyroscope-only method. For each method, the “Training Data” column indicates the amount of training data used per activity, and whether linear interpolation was applied to the data. For each participant’s training data, we used 5 s per activity, with the exceptions of the sit-to-stand and stand-to-sit transitions. The “Test Data” column indicates whether linear interpolation was applied to the test data.

Sensor	Training Data	Test Data
Joint-sensor	Accelerometer: 5 s of the	Accelerometer:
	original training data for each activity	original test data
	Gyroscope: 5 s of the training	Gyroscope:
	data for each activity, interpolated	test data interpolated to
	to the accelerometer timestamps	the accelerometer timestamps
Accelerometer only	5 s of the original training	Original test data
	data for each activity	
Gyroscope only	5 s of the original training	Original test data
	data for each activity	

**Table 2:** Amount of test data per participant. This table shows the total number of samples per participant across steps 1, 2, 3, 5, and 6 of the test data collection. The sampling frequency was approximately 10 Hz (i.e., 10 samples per second). For each participant, we present the number of samples of each activity type. The activity types observed in the test data collection included walking (“walk”), standing (“stand”), ascending stairs (“stair up”), descending stairs (“stair down”), sitting (“sit”), the sit-to-stand transition (“sit-to-stand”), the stand-to-sit transition (“stand-to-sit”), and going through a revolving door (“revolving door”). The “walk” activity includes slow-, normal-, and fast-paced walking.

	Participant 1	Participant 2	Participant 3	Participant 4
walk	3288	3135	3287	3246
stand	201	150	240	203
stair up	321	302	361	342
stair down	404	274	324	302
sit	291	232	242	251
sit-to-stand	21	20	20	20
stand-to-sit	20	20	20	20
revolving door	50	31	20	30

**Table 3:** Average accuracy of each method for different activity groups. The vigorous activities included walking, ascending stairs, and descending stairs. The stationary activities included sitting and standing. The transition activities included stand-to-sit and sit-to-stand.

	Participant	Accelerometer	Gyroscope	Joint-Sensor
(A) All Activities	1	78.9	80.5	88.9
	2	68.7	62.4	80.2
	3	71.3	74.6	78.1
	4	68.4	67.8	73.7
(B) Vigorous Activities	1	81.4	92.1	89.4
	2	67.1	82.6	82.8
	3	89.8	87.4	87.9
	4	79.2	87.7	89.2
(C) Stationary Activities	1	98.1	53.2	96.5
	2	97.2	47.0	96.4
	3	94.8	74.9	96.5
	4	90.6	63.4	89.0
(D) Transition Activities	1	56.0	90.2	80.5
	2	42.5	47.5	60.0
	3	20.0	55.0	45.0
	4	30.0	42.5	35.0

## Data Availability

The data presented in this study are openly available on Zenodo at 10.5281/zenodo.3925679 [26].

## Supplementary Materials

The following supporting information can be downloaded at: https://www.mdpi.com/article/10.3390/s22072618/s1, Figure S1: Step 1 for All Participants; Figure S2: Step 2 for All Participants; Figure S3: Step 3 (Normal Walking) for All Participants. Figure S4: Step 3 (Fast Walking) for All Participants. Figure S5: Step 3 (Slow Walking) for All Participants. Figure S6: Step 5 for All Participants. Figure S7: Step 6 (Ascending Stairs) for All Participants. Figure S8: Step 6 (Descending Stairs) for All Participants.
