# Supplementary material for: Smartphone-Based Activity Recognition Using Multistream Movelets Combining Accelerometer and Gyroscope Data
Source: Sensors (Basel). 2022 Mar 29;22(7):2618. doi: 10.3390/s22072618 (PMC9002497; doi:10.3390/s22072618)
Supplement: Supplementary file 1 [file sensors-22-02618-s001.zip › sensors-1606531-supplementary.pdf]

Supplement for "Smartphone-Based Activity Recognition using Multistream Movelets Combining Accelerometer and Gyroscope Data"

Emily J. Huang[1], Kebin Yan[2], and Jukka-Pekka Onnela[3]

[1]Department of Mathematics and Statistics, Wake Forest University
[2]Department of Biostatistics, Epidemiology, and Informatics, University of Pennsylvania
[3]Department of Biostatistics, Harvard University

March 26, 2022

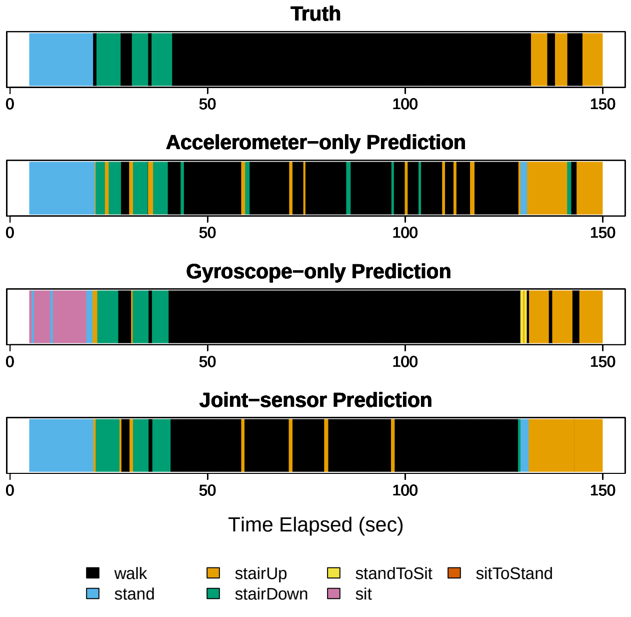

(A) Participant 1

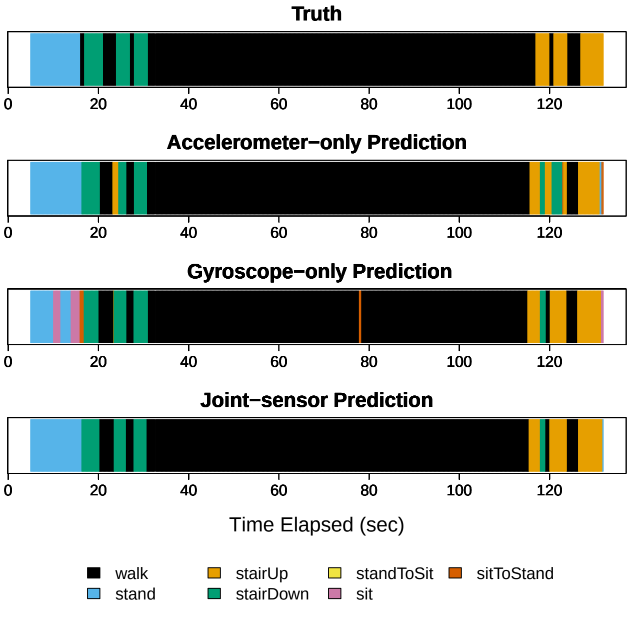

(B) Participant 2

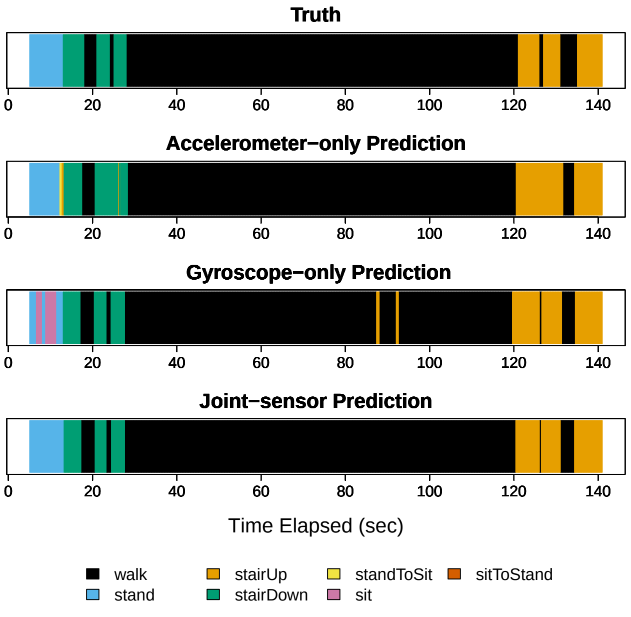

(C) Participant 3

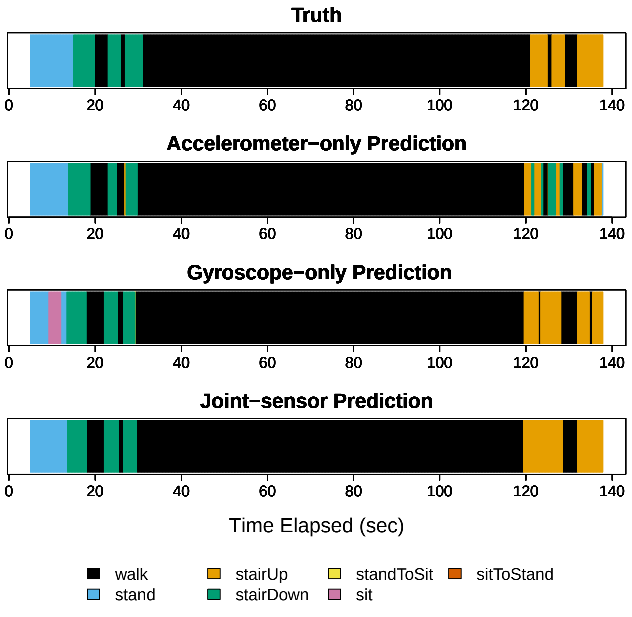

(D) Participant 4

Figure S1: Step 1 for All Participants. *This figure shows the results for Step 1. Panel A of the figure corresponds to Participant 1, Panel B to Participant 2, Panel C to Participant 3, and Panel D to Participant 4. In each panel, the top row shows the ground truth activity labels based on video footage. The second row shows the activity classifications from the accelerometer-only method, the third row shows the activity classifications from the gyroscope-only method, and the fourth row shows the activity classifications from the joint-sensor method.*

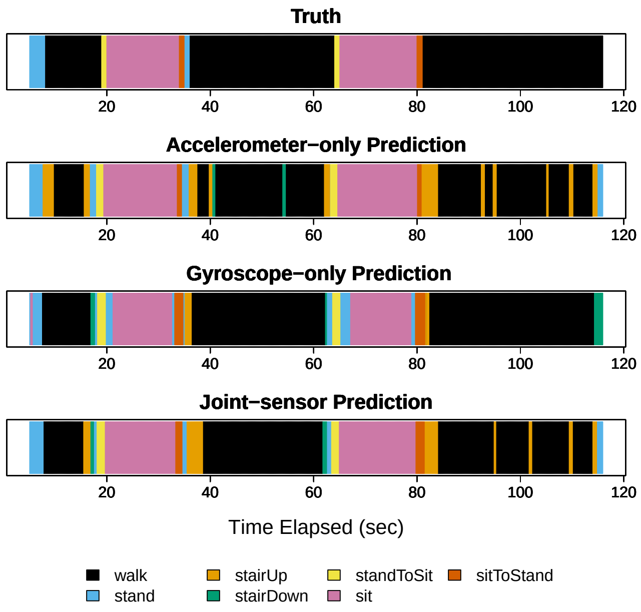

(A) Participant 1

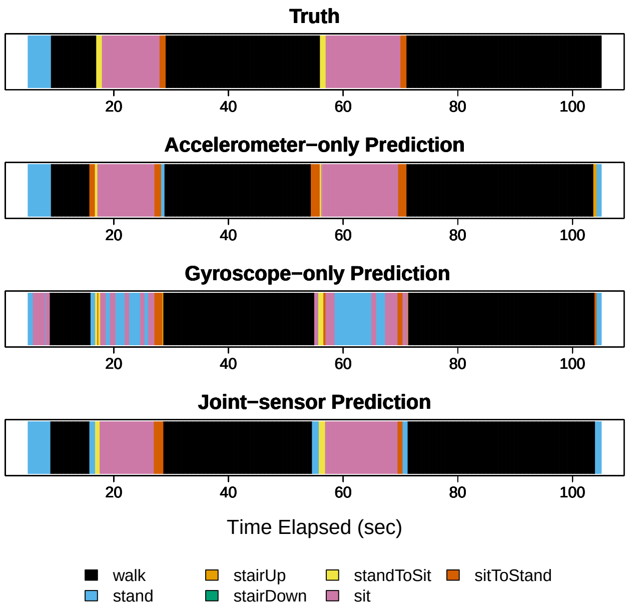

(B) Participant 2

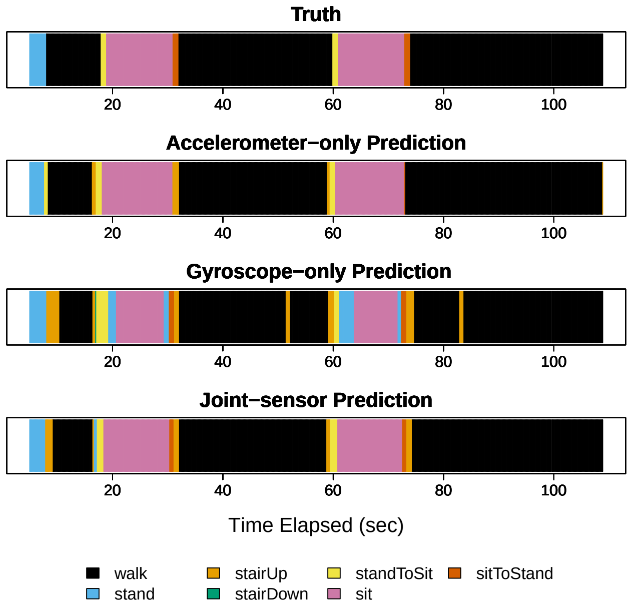

(C) Participant 3

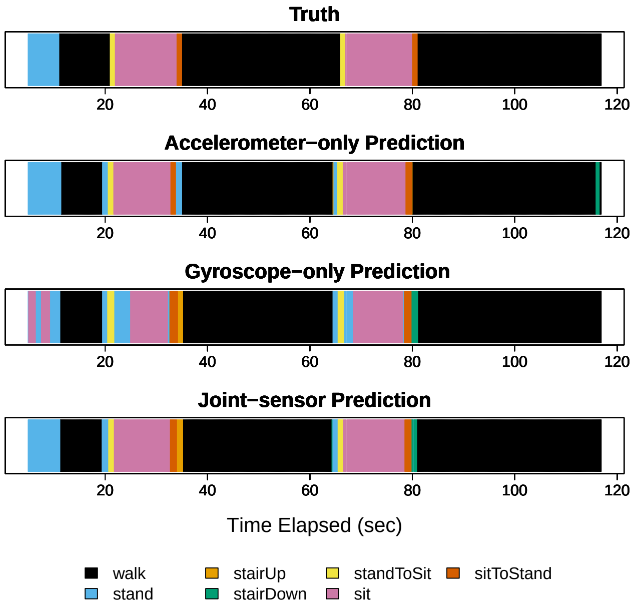

(D) Participant 4

Figure S2: Step 2 for All Participants. *This figure shows the results for Step 2. See Figure S1 for details on the formatting of the figure.*

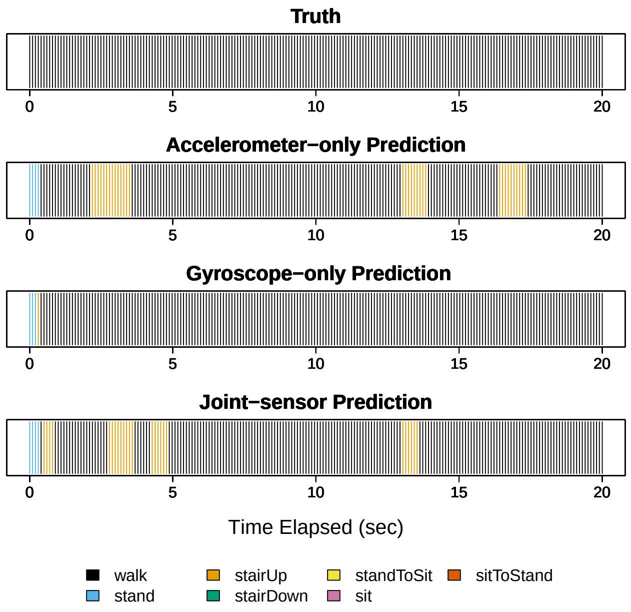

(A) Participant 1

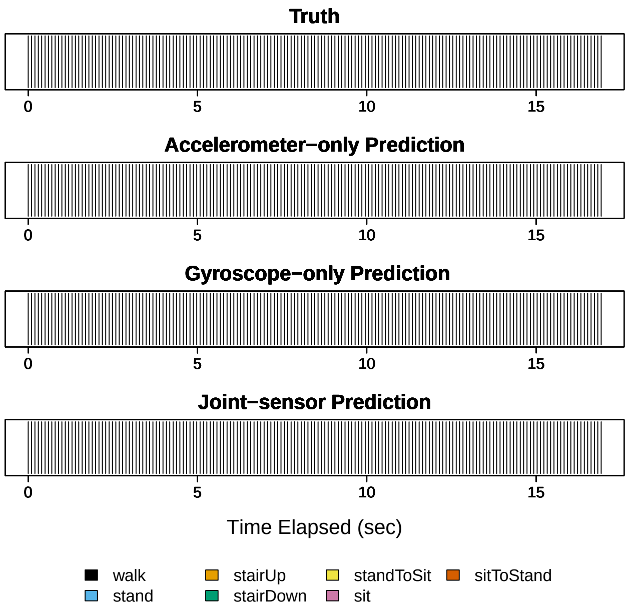

(B) Participant 2

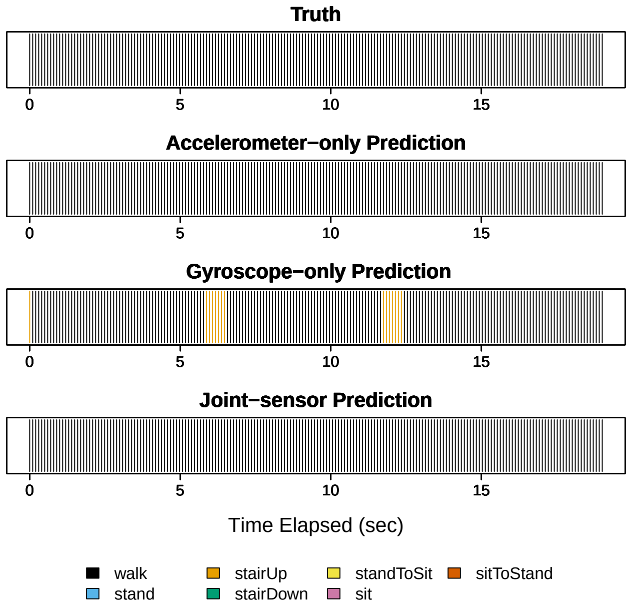

(C) Participant 3

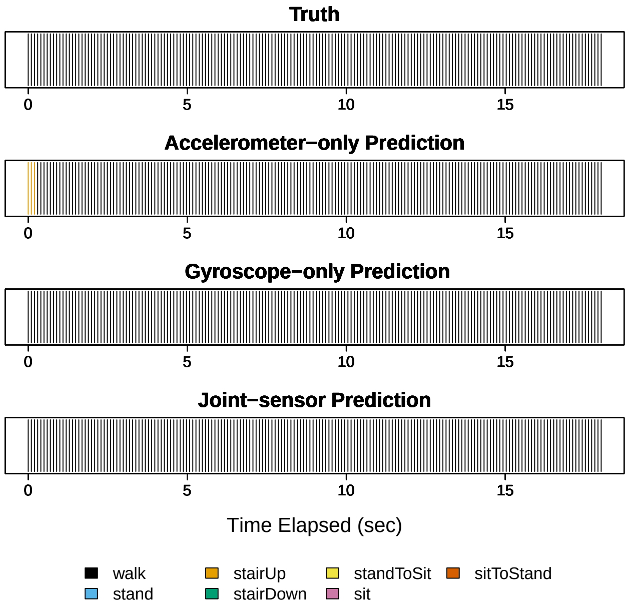

(D) Participant 4

Figure S3: Step 3 (Normal Walking) for All Participants. *This figure shows the results for the normal walking portion of Step 3. See Figure S1 for details on the formatting of the figure.*

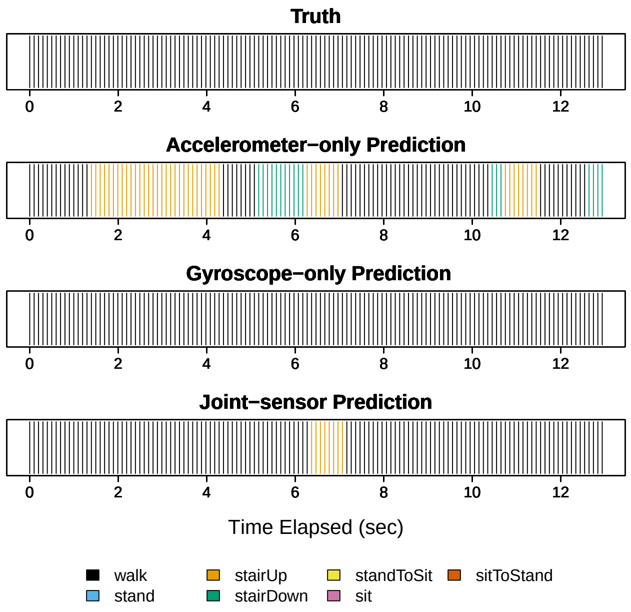

(A) Participant 1

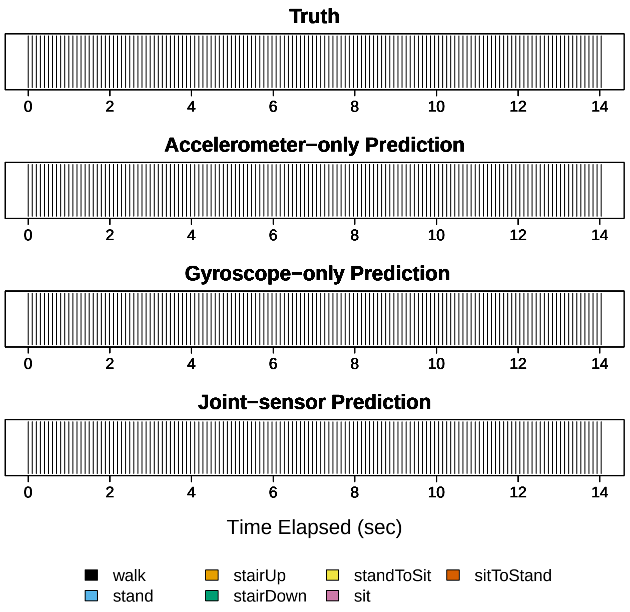

(B) Participant 2

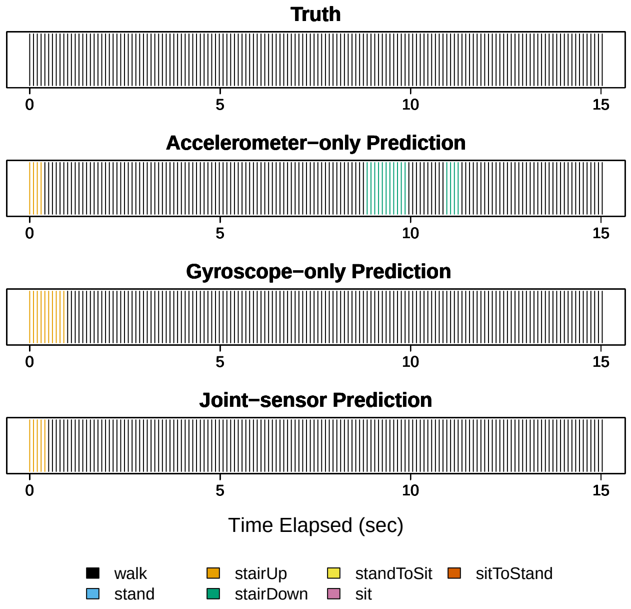

(C) Participant 3

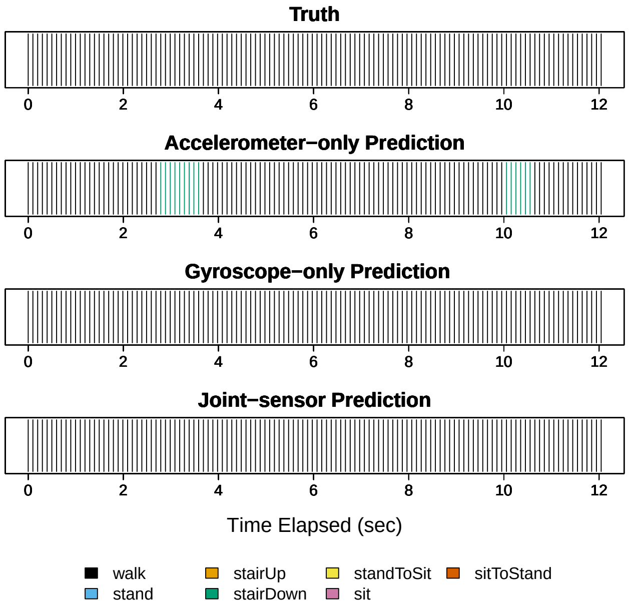

(D) Participant 4

Figure S4: Step 3 (Fast Walking) for All Participants. *This figure shows the results for the fast walking portion of Step 3. See Figure S1 for details on the formatting of the figure.*

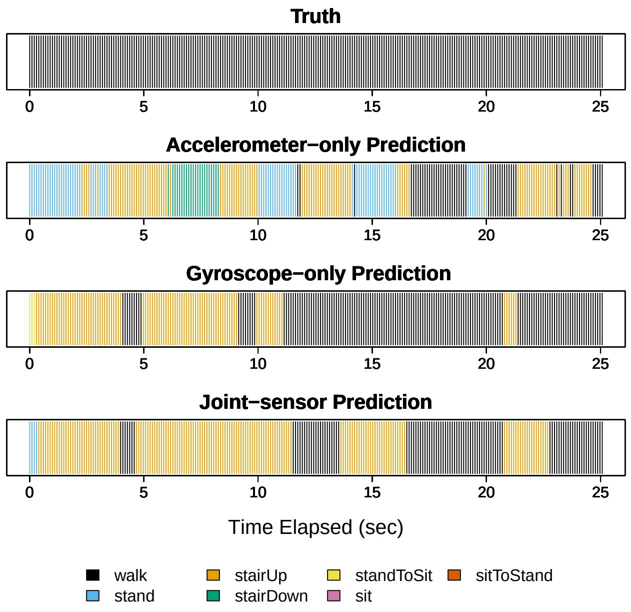

(A) Participant 1

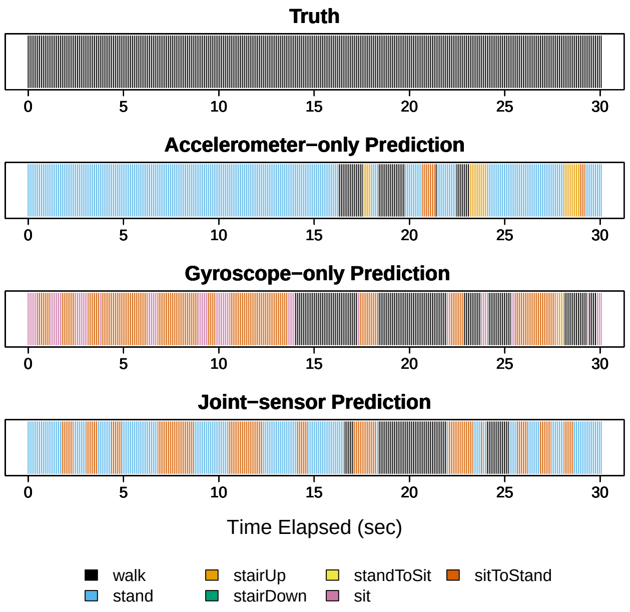

(B) Participant 2

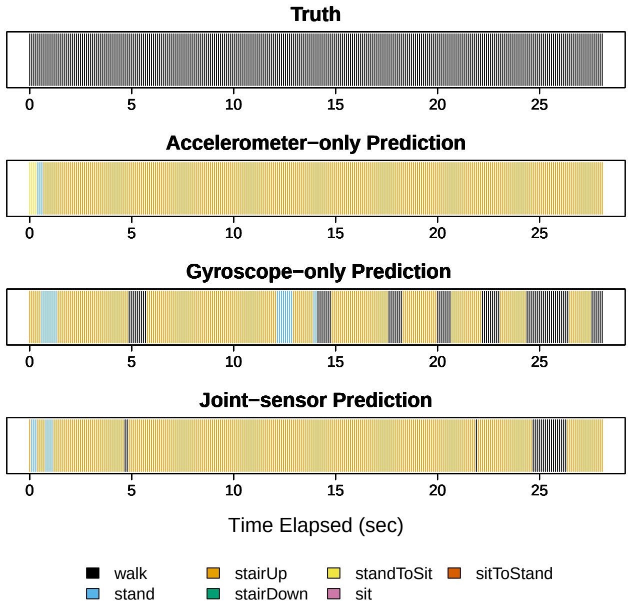

(C) Participant 3

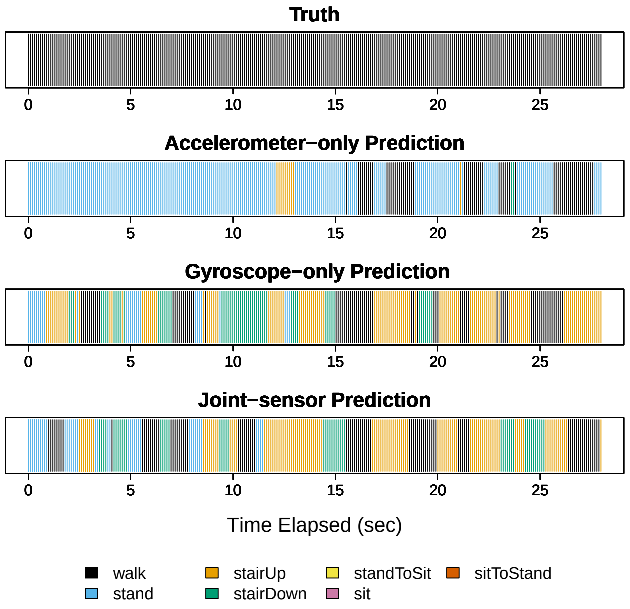

(D) Participant 4

Figure S5: Step 3 (Slow Walking) for All Participants. *This figure shows the results for the slow walking portion of Step 3. See Figure S1 for details on the formatting of the figure.*

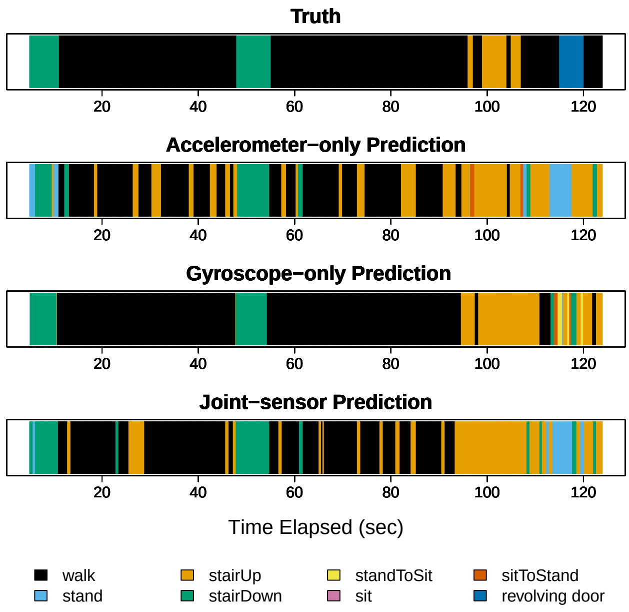

(A) Participant 1

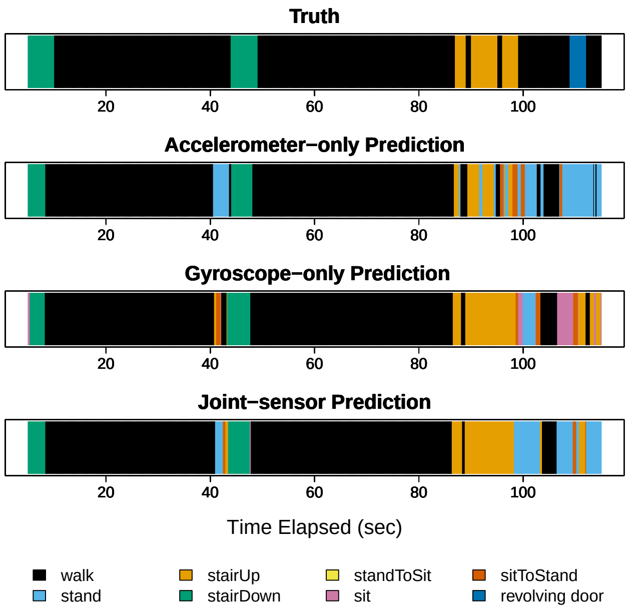

(B) Participant 2

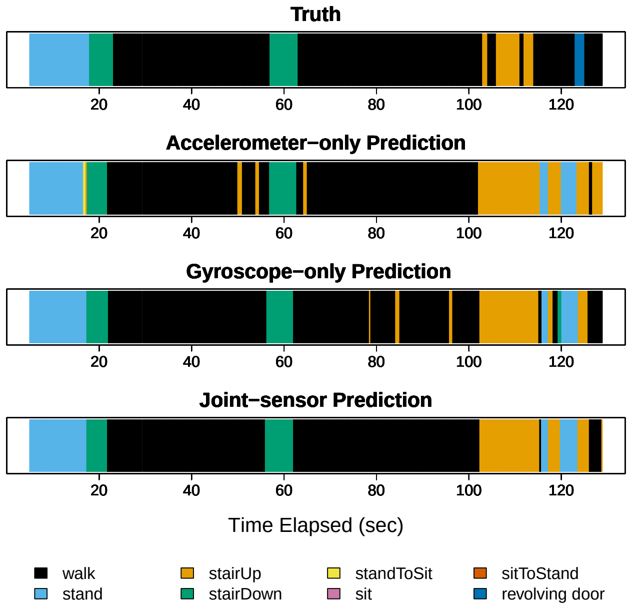

(C) Participant 3

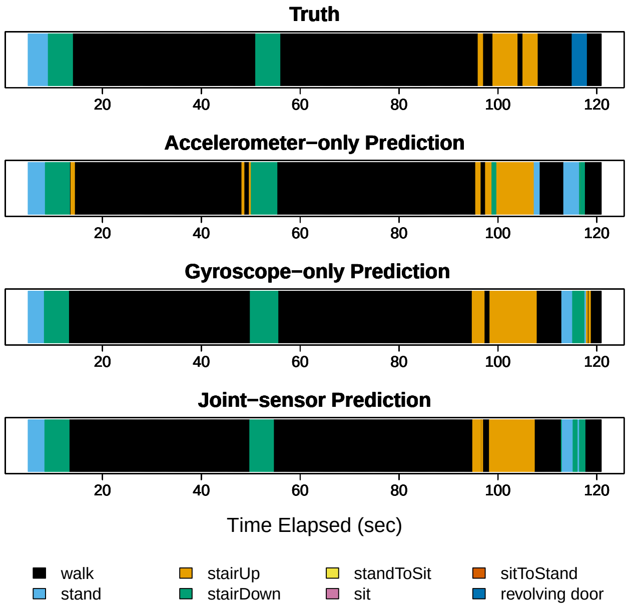

(D) Participant 4

Figure S6: Step 5 for All Participants. *This figure shows the results for Step 5. See Figure S1 for details on the formatting of the figure.*

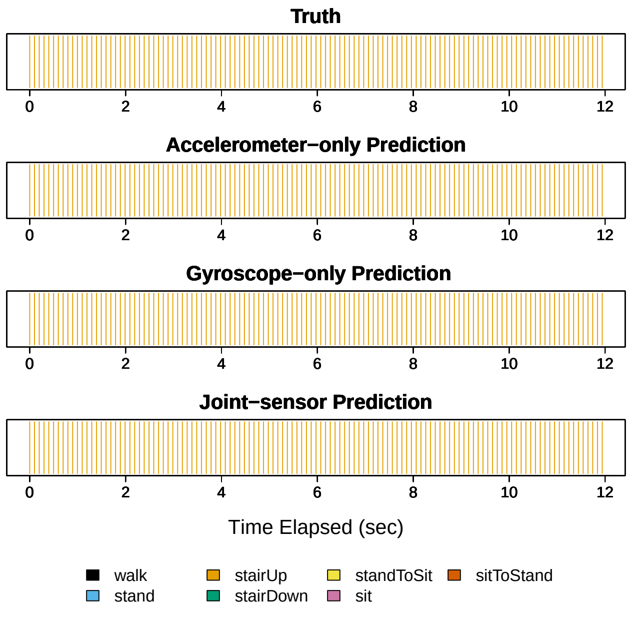

(A) Participant 1

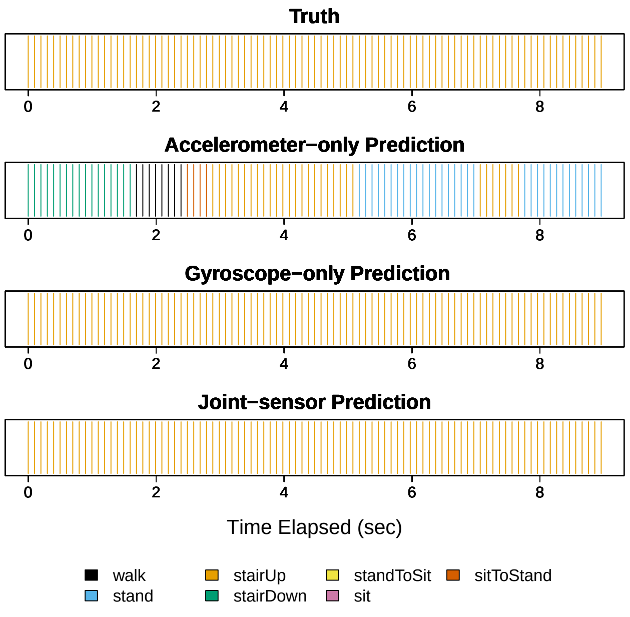

(B) Participant 2

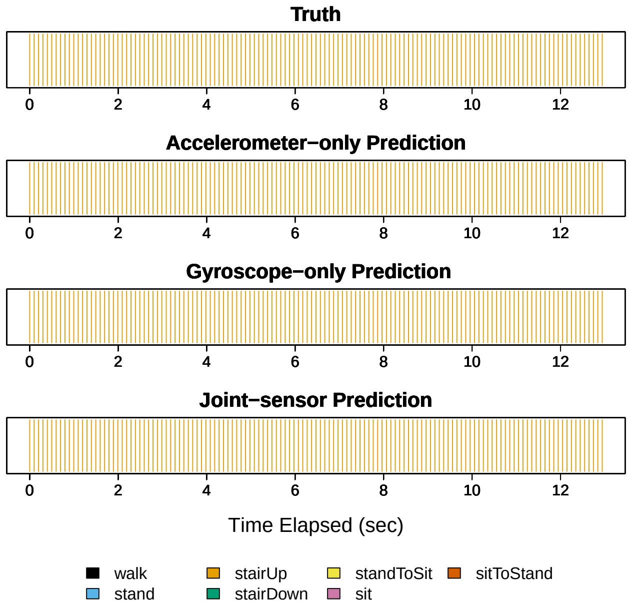

(C) Participant 3

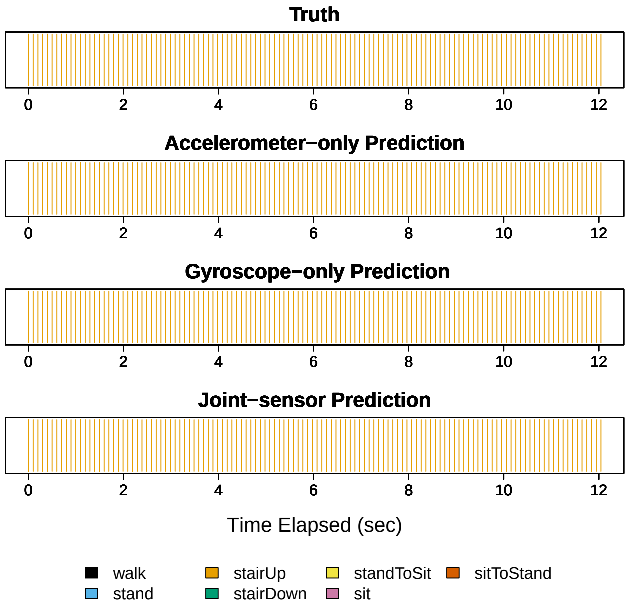

(D) Participant 4

Figure S7: Step 6 (Ascending Stairs) for All Participants. *This figure shows the results for the ascending stairs portion of Step 6. See Figure S1 for details on the formatting of the figure.*

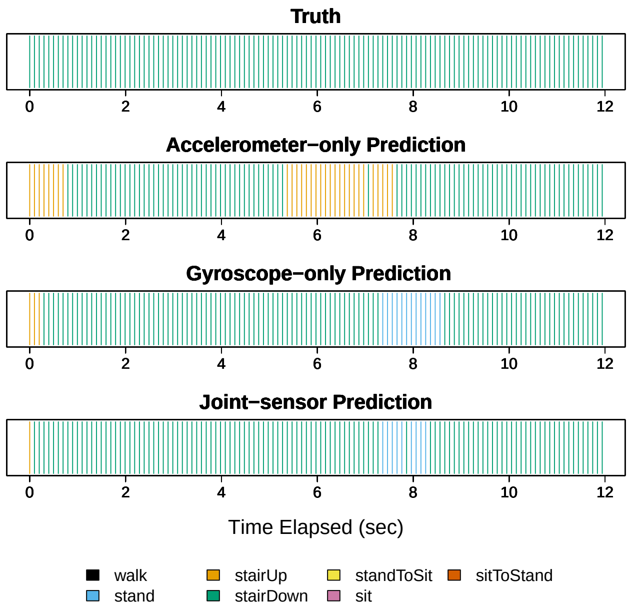

(A) Participant 1

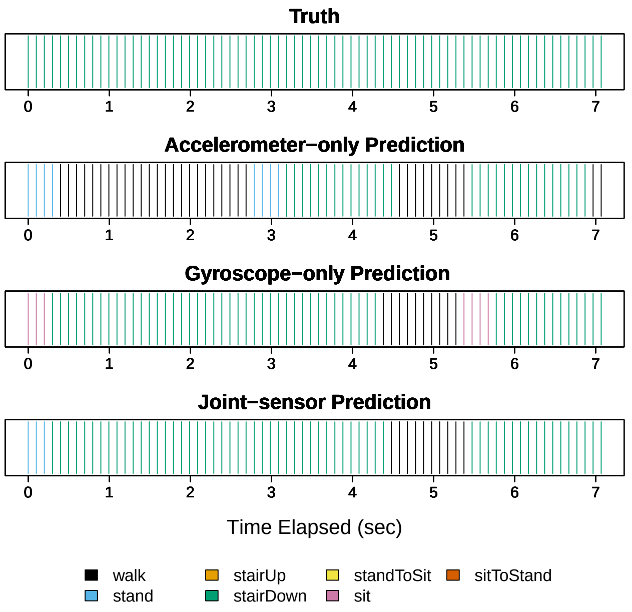

(B) Participant 2

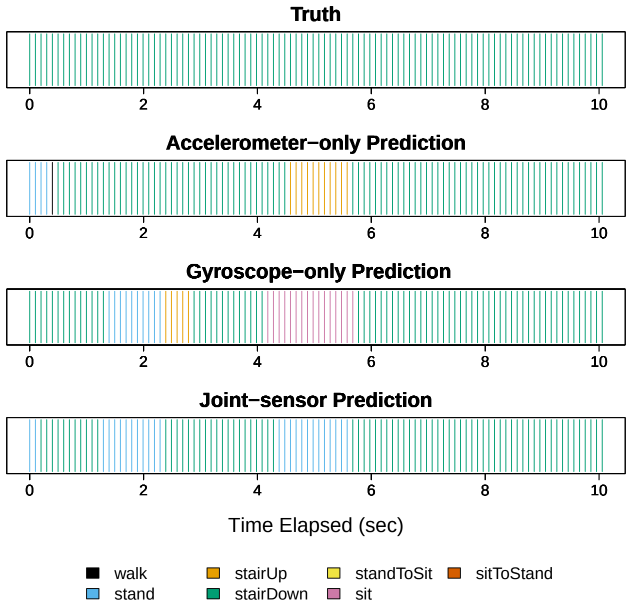

(C) Participant 3

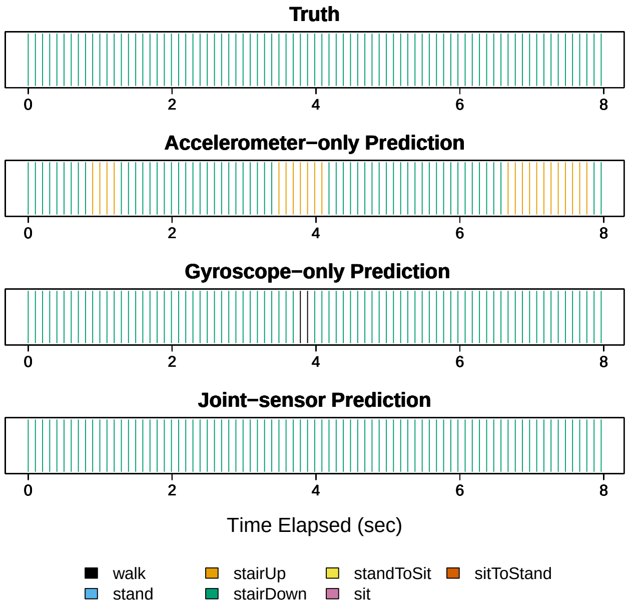

(D) Participant 4

Figure S8: Step 6 (Descending Stairs) for All Participants. *This figure shows the results for the descending stairs portion of Step 6. See Figure S1 for details on the formatting of the figure.*
